# P-590. Impact of Pharmacoenhancers on the Pharmacokinetics and Safety of Lenacapavir in People with HIV

**DOI:** 10.1093/ofid/ofae631.788

**Published:** 2025-01-29

**Authors:** Vamshi Jogiraju, Naveed A Shaik, Furong Wang, Hui Wang, Hadas Dvory-Sobol, Ramesh Palaparthy, Renu Singh

**Affiliations:** Gilead Sciences, Inc., Foster City, California; Gilead Sciences, Inc., Foster City, California; Gilead Sciences, Inc., Foster City, California; Gilead Sciences Inc., Foster City, California; Gilead Sciences, Foster City, California; Gilead Sciences Inc, Foster City, CA; Gilead Sciences Inc, Foster City, CA

## Abstract

**Background:**

Lenacapavir (LEN) is approved for treatment of multidrug-resistant HIV-1 in combination with other antiretrovirals in heavily treatment-experienced (HTE) people with HIV (PWH). In the pivotal Phase 2/3 CAPELLA study (NCT04150068), participants (N=72) received oral LEN loading doses (Days 1 and 2: 600 mg; Day 8: 300 mg), followed by a 927 mg subcutaneous (SC) maintenance dose given every 6 months (Q6M) starting from Day 15. LEN is a substrate of P-gp (P-glycoprotein), CYP (Cytochrome P450) 3A and UGT (UDP-glucuronosyltransferase) 1A1; pharmacoenhancers/boosters such as cobicistat and ritonavir inhibit P-gp and CYP3A and are likely to affect LEN PK. As boosted protease inhibitors are commonly used in HTE PWH, we evaluated the impact of pharmacoenhancers on LEN PK and safety in PWH from the CAPELLA study.

**Figure 1:**
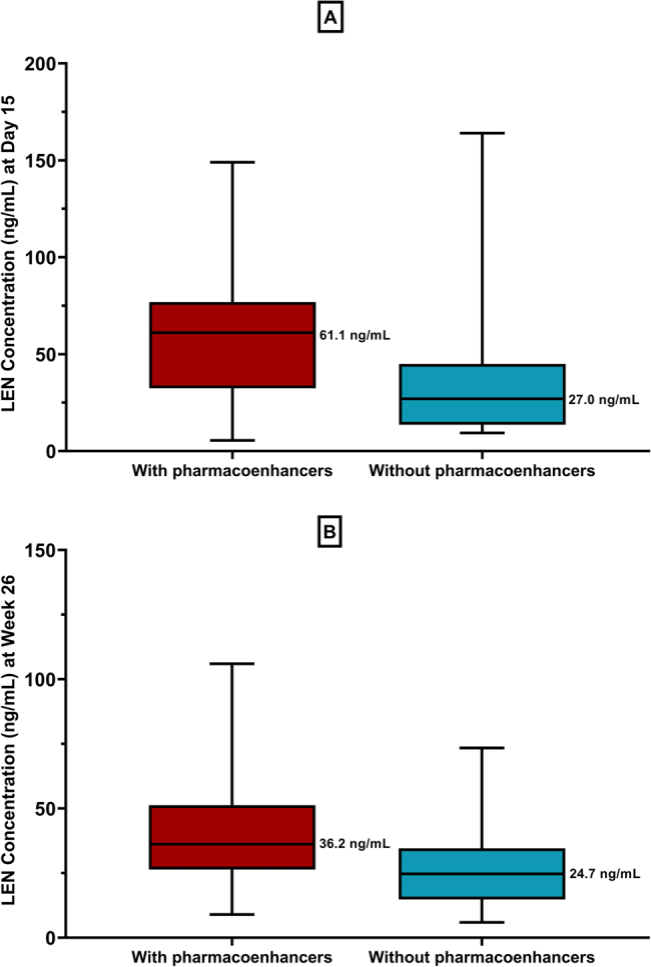
LEN Ctrough following (A) oral loading period (Day 15), and (B) SC injection (Week 26), with and without pharmacoenhancers

Horizontal lines of the box represent the median with its interquartile, and error bars represent the range. Median values are indicated in individual plots.

**Methods:**

Sparse PK samples were collected during the oral loading and the maintenance periods in the CAPELLA study. LEN plasma concentrations were summarized in participants receiving background regimens with or without pharmacoenhancers, at the end of oral loading period on Day 15 (N=45 and N=27; respectively) and at the end of first dosing interval at Week 26 (N=42 and N=30; respectively). Treatment emergent adverse events (AE) were assessed among participants receiving background regimens with or without pharmacoenhancers.

**Results:**

Following oral loading of LEN (Days 1, 2 and 8), Day 15 median C_trough_ were 61.1 and 27.0 ng/mL with and without pharmacoenhancers, respectively. Following SC dose on Day 15, median C_trough_ at the end of Week 26 were 36.2 ng/mL and 24.7 ng/mL with and without pharmacoenhancers, respectively. The safety profile of LEN with or without pharmacoenhancers was generally similar (respectively: LEN-related AEs, 61.9% vs. 63.3%).

**Conclusion:**

Pharmacoenhancers led to a modest increase in LEN exposure, which was not considered clinically relevant. The safety profile of LEN with or without pharmacoenhancers was similar. LEN can be coadministered with background regimens containing pharmacoenhancers without dose adjustment.

**Disclosures:**

**Vamshi Jogiraju, PhD**, Gilead Sciences, Inc.: Employee|Gilead Sciences, Inc.: Stocks/Bonds (Public Company) **Naveed A. Shaik, PhD**, Gilead Sciences, Inc.: Employee|Gilead Sciences, Inc.: Stocks/Bonds (Public Company) **Furong Wang, n/a**, Gilead Sciences, Inc.: Employee|Gilead Sciences, Inc.: Stocks/Bonds (Public Company) **Hui Wang, PhD**, Gilead Sciences, Inc.: Employee|Gilead Sciences, Inc.: Stocks/Bonds (Public Company) **Hadas Dvory-Sobol, PhD**, Gilead Sciences, Inc.: Employee|Gilead Sciences, Inc.: Stocks/Bonds (Public Company) **Ramesh Palaparthy, PhD**, Gilead Sciences, Inc.: 1475-US-PSP, 1475-WO-PCT, 1474-US-PSP, 1515-US-PSP, 1515-WO-PCT|Gilead Sciences, Inc.: Employee|Gilead Sciences, Inc.: Stocks/Bonds (Public Company) **Renu Singh, PhD**, Gilead Sciences, Inc.: 1475-US-PSP, 1475-WO-PCT, 1474-US-PSP, 1515-US-PSP, 1515-WO-PCT|Gilead Sciences, Inc.: Employee|Gilead Sciences, Inc.: Stocks/Bonds (Public Company)

